# Supplementary material for: The Effects of a Partially Hydrolyzed Formula with Low Lactose and Probiotics on Mild Gastrointestinal Disorders of Infants: A Single-Armed Clinical Trial
Source: Nutrients. 2021 Sep 25;13(10):3371. doi: 10.3390/nu13103371 (PMC8540403; doi:10.3390/nu13103371)
Supplement: Supplementary file 1 [file nutrients-13-03371-s001.zip › nutrients-1385782-supplementary.pdf]

Supplementary data

Table S1. Composition of study formula

Nutrition	Composition
Energy (kcal/100mL)	280
Protein (g/100mL)	1.27
Lipids (g/100mL)	3.4
Carbohydrates (g/100mL)	7.83
**Lactose (g/100mL)**	<3.1
Vitamins	
A (μg RE/100mL)	66.66
D (μg/100mL)	0.89
E (mg α-TE/100mL)	1.31
K_1 (μg/100mL)	5.75
B_1 (μg/100mL)	65.35
B_2 (μg/100mL)	117.63
B_6 (μg/100mL)	47.05
B_{12} (μg/100mL)	0.144
C (mg/100mL)	9.02
Minerals	
Sodium (mg/100mL)	26.14
Magnesium	6.54
Potassium	73.85
Calcium (mg/100mL)	45.22
Iron (mg/100mL)	0.68
Zinc (mg/100mL)	0.65
**Bifidobacterium Bb-12 (CFU/g)**	$>10^6$

Table S2. Demographic characteristics and IGSQ scores at baseline for the included and excluded participants

	Included (n=80)	Excluded (n=12)	*P* value
Sex			
Boy	39 (48.8)	5 (41.7%)	0.882
Girl	41 (51.3)	7 (58.3%)	
Age (month)	2.0 ± 1.5	1.8 ± 1.6	0.746
Delivery mode			
Vaginal delivery	50 (62.5)	8 (66.7%)	1.000
Cesarean delivery	30 (37.5)	4 (33.3%)	
Birth weight (g)	3266 ± 470	3400 (±380)	0.350
Feeding Practice			
Mixed feeding	78 (97.5)	12 (100.0%)	1.000
Exclusive formula feeding	2 (2.5)	0 (0.0%)	
IGSQ scores at baseline	36.0 ± 5.7	36.2 ± 7.4	0.222
Maternal age (year)	32.1 ± 5.3	30.0 (4.5)	0.200
Paternal age (year)	32.5 ± 5.8	32.5 (5.8)	0.193
Maternal education*			
College or above	46 (58.2)	6 (50.0)	
Senior high school	25 (31.6)	3 (25.0)	0.396
Junior high school or below	8 (10.1)	3 (25.0)	
Paternal education*			
College or above	48 (60.8)	7 (58.3)	
Senior high school	23 (29.1)	3 (25.0)	0.776
Junior high school or below	8 (10.1)	2 (16.7)	

note: *Education of one participant's parents was unknown.

Table S3. Sleeping measures in the first three days of feeding intervention

Domain	Day 1	Day 2		Day 3	
	Mean ± SD/ Median (Range)	Mean ± SD / Median (Range)	MD (95%CI)/ RR (95% CI)	Mean ± SD / Median (Range)	MD (95%CI)/ RR (95% CI)
Sleeping hours	15.0 ± 3.9	15.1 ± 3.3	0.04 (-0.5, 0.6)	15.1 ± 3.2	0.1 (-0.4, 0.7)
Times of wake-ups at night	4 (0, 6)	3 (0, 8)	1.0 (0.8, 1.2)	3 (2, 4)	0.9 (0.8, 1.1)

Table S4. Infants' growth measures at Day 0 and Day 14 of feeding intervention

	Day 0<br>Mean ± SD	Day 14<br>Mean ± SD	*P* value
Height (cm)	58.6 ± 4.5	60.2 ± 4.4	<0.001
Z-score of height	0.22 ± 0.97	0.31 ± 1.00	0.079
Weight (g)	5374 ± 1405	5925 ± 1356	<0.001
Z-score of weight	-0.11 ± 1.00	0.13 ± 1.02	<0.001
Head circumference (cm)	38.5 ± 2.1	39.2 ± 2.0	<0.001
Z-score of head circumference	-0.31 ± 1.01	-0.28 ± 0.90	0.505
BMI (kg/m^2)	15.3 ± 2.0	16.1 ± 1.8	<0.001
Z-score of BMI	-0.37 ± 1.06	-0.09 ± 1.04	<0.001

Notes: Z-score of each parameter was calculated according to the WHO child growth standards (https://www.who.int/childgrowth/standards/en/).
